# Theoretical and Experimental Gas Volume Quantification of Micro- and Nanobubble Ultrasound Contrast Agents

**DOI:** 10.3390/pharmaceutics12030208

**Published:** 2020-03-01

**Authors:** Eric C. Abenojar, Ilya Bederman, Al C. de Leon, Jinle Zhu, Judith Hadley, Michael C. Kolios, Agata A. Exner

**Affiliations:** 1 Department of Radiology, Case Western Reserve University, 10900 Euclid Ave. Cleveland, OH 44106, USA; eca20@case.edu (E.C.A.); acd77@case.edu (A.C.d.L.); jxz884@case.edu (J.Z.); 2 Department of Genetics and Genome Sciences, School of Medicine, Case Western Reserve University, 10900 Euclid Ave. Cleveland, OH 44106, USA; ilya@case.edu; 3 Malvern Panalytical, Westborough, MA 01581, USA; judy.hadley@malvern.com; 4 Department of Physics, Ryerson University, Toronto, ON M5B 2K3, Canada; mkolios@ryerson.ca; 5 Department of Biomedical Engineering, Case Western Reserve University, 10900 Euclid Ave. Cleveland, OH 44106, USA

**Keywords:** gas chromatography/mass spectrometry, contrast agents, ultrasound, nanobubble, microbubble, resonant mass measurement, dynamic light scattering, nanoparticle tracking analysis, coulter counter, perfluoropropane, gas volume

## Abstract

The amount of gas in ultrasound contrast agents is related to their acoustic activity. Because of this relationship, gas volume has been used as a key variable in normalizing the in vitro and in vivo acoustic behavior of lipid shell-stabilized bubbles with different sizes and shell components. Despite its importance, bubble gas volume has typically only been theoretically calculated based on bubble size and concentration that is typically measured using the Coulter counter for microbubbles and nanoparticle tracking analysis (NTA) for nanoscale bubbles. However, while these methods have been validated for the analysis of liquid or solid particles, their application in bubble analysis has not been rigorously studied. We have previously shown that resonant mass measurement (RMM) may be a better-suited technique for sub-micron bubble analysis, as it can measure both buoyant and non-buoyant particle size and concentration. Here, we provide validation of RMM bubble analysis by using headspace gas chromatography/mass spectrometry (GC/MS) to experimentally measure the gas volume of the bubble samples. This measurement was then used as ground truth to test the accuracy of theoretical gas volume predictions based on RMM, NTA (for nanobubbles), and Coulter counter (for microbubbles) measurements. The results show that the headspace GC/MS gas volume measurements agreed well with the theoretical predictions for the RMM of nanobubbles but not NTA. For nanobubbles, the theoretical gas volume using RMM was 10% lower than the experimental GC/MS measurements; meanwhile, using NTA resulted in an 82% lower predicted gas volume. For microbubbles, the experimental gas volume from the GC/MS measurements was 27% lower compared to RMM and 72% less compared to the Coulter counter results. This study demonstrates that the gas volume of nanobubbles and microbubbles can be reliably measured using headspace GC/MS to validate bubble size measurement techniques. We also conclude that the accuracy of theoretical predictions is highly dependent on proper size and concentration measurements.

## 1. Introduction

Ultrasound (US) is a noninvasive, safe, accessible, and inexpensive medical imaging modality widely used around the world. Echogenic bubble contrast agents are used in US imaging to improve soft tissue contrast, leading to more accurate diagnosis in different clinical applications, from cardiovascular imaging and echocardiography to tumor imaging [1]. There are currently three commercially available microbubble contrast agents approved by the Food and Drug Administration (FDA) for clinical use: Optison (GE Healthcare) and Definity (Lantheus), which are protein- or phospholipid-shell stabilized microbubbles of perfluoropropane (C_3_F_8_) gas, and Lumason/Sonovue (Bracco), which are lipid-shell stabilized sulfur hexafluoride (SF_6_) bubbles [2]. While microbubbles are a robust intravascular blood pool contrast agent [3,4], they have shown short circulation times (on the order of 2–10 min) arising from the dissolution of the gas into the surrounding medium. In addition, because of their 1–10 µm diameter, the microbubbles remain in the vasculature, and, thus, have limited applications. Recently, significant effort has been devoted to developing new generations of more stable bubbles in an expanded range of sizes, including in the nanoscale [5,6,7]. Stable sub-micron or nanobubbles extend bubble circulation time and have been shown to localize beyond the tumor vasculature via the enhanced permeability effect (EPR) [8,9], hence offering many new potential ultrasound contrast agent (UCA) applications for tumor imaging and therapy.

The acoustic response and longevity of bubble UCAs depend on a multitude of formulation factors, with two of the most important being shell composition and the type of gas used. In an acoustic field, the bubble contracts and expands, and the longevity of the bubble is determined by the flexibility and strength of its shell [10,11,12,13]. The type of gas used dictates the ease with which it diffuses out of the bubble, which eventually leads to bubble collapse. Hydrophobic gases are typically employed because they are immiscible to the aqueous environment where UCAs are clinically used, which prevents it from leaking out fast, leading to a longer bubble half-life. The type of gas used to prepare the bubble also has a large influence on its stability [14,15]. Bubbles filled with air (where nitrogen is the main component) are less stable than those prepared with a hydrophobic gas (e.g., C_3_F_8_, SF_6_). Hydrophobic gases are less prone to diffuse out of the bubble due to their incompatibility with the hydrophilic liquid matrix. Among hydrophobic gases, those with higher molecular weight, higher gas density, and low diffusivity coefficient are expected to give more stable bubbles [16]. Perfluorocarbons (PFCs) are biocompatible, biologically inert, and highly stable chemicals that are not metabolized in the body after injection [17,18]. Therefore, they are the most ideal gas to use for preparing highly stable, clinically-safe bubble UCAs. In addition, increasing the chain length of perfluorocarbons by –CF_2_ leads to an order of magnitude decrease in solubility in water [16,19]. Thus, a lot of research has also focused on using heavier perfluorocarbons, such as C_4_F_10_, C_5_F_12_, and C_6_F_14_ [20]. PFCs have also been shown to reduce interfacial tension, which can further improve bubble performance [21,22].

Several reports have used total bubble gas volume as the main parameter in evaluating bubbles with the same shell components for ultrasound activity rather than the bubble count [23,24,25]. Microbubbles of different sizes evaluated with a similar gas volume were reported to show a similar circulation half-life, which suggests that gas volume may be a better predictor of UCA stability than bubble size [26]. A similar dependence on microbubble gas volume and not on size or size distribution was observed for blood brain barrier (BBB) opening using focused ultrasound [27]. Nanobubbles with similar gas volumes to commercially available micron-sized UCAs, Definity and Optison, were shown to be more reliable for BBB opening, which could be a result of its smaller size and larger number of bubbles present [28]. A recent report by our group showed that at similar gas volumes, nanobubbles are less affected by changes in gas volume concentration compared to microbubbles [25].

Bubble gas volume is typically estimated theoretically by assuming a bubble sphere, calculating its volume, and subtracting the bubble shell volume accordingly [26,27,28]. For a phospholipid bubble, the theoretical amount of gas is typically quantified by taking into account that the thickness of the bubble shell is 2.5 nm (typical lipid bilayer thickness is 5 nm and a bubble shell is a lipid monolayer) [29]. The volume is typically calculated based on microbubble size and concentration measurements from the Coulter counter (Beckman Coulter, Indianapolis, IN, USA) [27,30]. Most Coulter counter instruments used have a limit of detection of 600 nm (with the lowest at 200 nm) [31,32] and suffer from documented coincidence errors, which may reduce the accuracy of the measurement, especially for polydisperse bubble populations. For sub-micron bubbles, the size may be measured by dynamic light scattering (DLS), while both size and concentration can be determined by nanoparticle tracking analysis (NTA). It is also important to note that none of these techniques are capable of distinguishing between buoyant (bubble) and non-buoyant populations of a sample, limiting their accuracy in providing accurate bubble concentrations [33]. To overcome these drawbacks, a resonant mass measurement (RMM) technique has been used to quantify the size and concentration of the bubbles separately from non-buoyant particles [25,33,34,35,36,37] and to determine the theoretical gas volume in a nanobubble population. However, these theoretical calculations are rarely verified experimentally, despite their importance in evaluating and comparing bubbles. Actual gas volume measurements using headspace gas chromatography/mass spectrometry (GC/MS) have rarely been performed [38] or correlated with theoretical measurements [25].

In our prior work [26], we utilized headspace GC/MS to experimentally measure bubble gas volume. These results were compared to the theoretical quantification of bubble gas volume using the size and concentration obtained from RMM. Here, we aim to apply the same technique to carry out a comprehensive validation analysis of the various methods used to experimentally and theoretically quantify bubble gas volume. Specifically, we expand on our previous work [26] in two ways: firstly, testing the effect of bubble volume used for headspace GC/MS analysis was evaluated, where different bubble volumes (six sampling volumes from 50–500 µL for nanobubbles (NBs) and four sampling volumes from 200–500 µL for microbubbles (MBs)) were analyzed independently and in triplicate using headspace GC/MS. Our previous work used a single bubble volume for the experimental gas measurement of both NBs and MBs. Secondly, comparing the theoretical quantification of bubble gas volume was measured using different techniques: nanoparticle tracking analysis (NTA) for NBs, Coulter counter for MBs, which is widely used in literature, and RMM for both NBs and MBs, which is a newer technique capable of simultaneously distinguishing between buoyant (bubble) and non-buoyant populations in a sample. In addition, the mean size obtained for these aforementioned techniques was also compared against DLS measurement. We then discuss the results and demonstrate the reliability of headspace GC/MS for experimental gas volume quantification and show which size and concentration characterization technique gives more reliable results compared to headspace GC/MS measurements.

## 2. Materials and Methods

### 2.1. Materials

The lipids 1,2-dibehenoyl-*sn*-glycero-3-phosphocholine (DBPC), 1,2 dipalmitoyl-*sn*-glycero-3-phosphate (DPPA), and 1,2-dipalmitoyl-*sn*-glycero-3-phosphoethanolamine (DPPE) were obtained from Avanti Polar Lipids (Pelham, AL, USA), and 1,2-distearoyl-*sn*-glycero-3-phosphoethanolamine-*N*-[methoxy(polyethylene glycol)-2000] (ammonium salt) (mPEG–DSPE) was obtained from Laysan Lipids (Arab, AL, USA). Propylene glycol was purchased from Sigma Aldrich (Milwaukee, WI, USA). Glycerol (99+%, Acros Organics, Morris Plains, NJ, USA) and phosphate-buffered saline solution (PBS, Gibco, Life Technologies, Waltham, MA, USA) were purchased from Fisher Scientific (Pittsburgh, PA, USA). Perfluoropropane (C_3_F_8_) was obtained from AirGas (Cleveland, OH, USA).

### 2.2. Bubble Formulation

The bubbles were prepared as previously reported [10]. Briefly, the DBPC, DPPE, DPPA, and mPEG–DSPE were dissolved in propylene glycol by sonication and heating at 80 °C. A solution of phosphate-buffered saline (PBS) (pH 7.4) and glycerol pre-heated at 80 °C was then added to the lipid solution and sonicated at room temperature for 10 min. This solution was transferred to a 3 mL headspace vial, capped with a rubber stopper, and sealed with an aluminum cap. Following purging of the vial with C_3_F_8_ gas, the bubbles were activated by mechanical agitation using a VialMix (Bristol–Myers Squibb Medical Imaging, Inc., N. Billerica, MA, USA) shaker for 45 s. The nanobubbles (NBs) were isolated from the bubble population based on their buoyancy by centrifugation at 50 *g* for 5 min [39]. Microbubble (MB) isolation was performed by diluting the bubbles with 10% *v/v* glycerol/propylene glycol in PBS, and centrifuging at 300 rcf for 10 min, after which the infranatant (liquid suspension) was discarded. The MB cake was re-dispersed in PBS and centrifuged at 30 rcf for 1 min, after which the cake was discarded and the infranatant collected. This process was repeated at centrifugal speeds of 70, 160, and 270 rcf for 1 min each, and finally at 300 rcf for 10 min [40]. At the last centrifugation step, the infranatant was discarded and the cake was re-dispersed in a solution of 10% *v/v* glycerol/propylene glycol in PBS. The sample was then transferred to a 3 mL headspace vial, capped with a rubber septum, and sealed with an aluminum cap. The vial was then flushed with C_3_F_8_ gas and stored in a refrigerator at 4 °C.

### 2.3. Size Characterization of Bubbles

The bubbles were characterized using four sizing techniques with different capabilities: (1) resonant mass measurement (RMM), (2) dynamic light scattering (DLS), (3) nanoparticle tracking analysis (NTA), and (4) Coulter counter. The RMM (Archimedes, Malvern Panalytical Inc., Westborough, MA, USA) could provide the particle’s mean size, size distribution, and concentration for both buoyant (bubble) and non-buoyant particle populations in a sample. Two RMM sensors with different limits of detection were used [37]: (1) a microsensor was used to characterize MBs, which could provide size measurements from 250 nm to 5 µm, and (2) a nanosensor was used to characterize the NBs, which provided measurements from 100 nm to 2 µm. The nanosensor and microsensor were calibrated with National Institute of Standards and Technology (NIST) traceable 565 nm and 994 nm polystyrene bead standards, respectively (ThermoFisher 4010S, Waltham MA, USA). The NBs and MBs were diluted in a 1:1000 and 1:500 ratio, respectively, with PBS (pH 7.4), to obtain a concentration of 10^8^ particles∙mL^−1^. This concentration resulted in an acceptable limit of detection (<0.02 Hz) and coincidence (<10%). A total of 1000 particles were measured for each trial performed (*n* = 3). The sensor and microfluidic tubing were cleaned with deionized water in between each run. Data was exported from the Archimedes software (version 1.2) and analyzed for positive and negative counts, which corresponded to buoyant (bubble) and non-buoyant particles, respectively. A density of 0.008 g∙mL^−1^ for positively buoyant particles and 1.3 g∙mL^−1^ for negatively buoyant particles was input into the Archimedes software to convert the measured mass to a particle diameter, which was based on the density of the perfluoropropane gas used to fill the bubbles and the density of vesicles, which had a similar structure and size to that of non-buoyant particles [41,42]. Dynamic light scattering (DLS) was used to measure the hydrodynamic diameter of the bubbles using a Litesizer™ 500 from Anton Paar, with a light source of 658 nm laser (40 mW) and a limit of detection of 0.3 nm. The samples were diluted 100-fold prior to measurement and three trials were performed for each sample type. This technique, however, could only provide for size measurement and not concentration. The nanoparticle tracking analysis (NTA) measurements were performed using a NanoSight NS300 (Malvern Panalytical Inc., Westborough, MA, USA) at Malvern Panalytical. The bubbles were shipped after isolation and freezing. A previous study by our group has shown that frozen nanobubbles exhibit preserved size distribution and acoustic properties for up to 4 weeks [43]. Finally, particle size and concentration were measured using a Beckman Multisizer 3 Coulter counter (Indianapolis, IN, USA) with an aperture of 20 µm and lower limit of detection of 400 nm, which is commonly used for bubble characterization [40]. A bubble sample of 10 µL was diluted in 20 mL of Isotone II in a 25 mL vial and then characterized with the Coulter counter. Sample measurements were performed in triplicate.

### 2.4. Quantification of Bubble Perfluoropropane Gas (C_3_F_8_) Volume via GC/MS

The perfluoropropane gas content of each bubble sample was obtained as previously described [25]. Briefly, the NB or MB solutions were placed in 2 mL headspace vials and sealed. The samples were sonicated at 50 °C for 20 min in order to destroy the bubbles and release the gas to the headspace of the vial using an ultrasonic bath (Branson Ultrasonics, Danbury, CT, USA) [38]. The relative concentration of C_3_F_8_ was determined using an Agilent 5977B-MSD equipped mass spectrometer with an Agilent 7890B gas chromatograph (GC/MS) system. A DB5-MS capillary column (30 m × 0.25 mm × 0.25 μm) was used with a helium flow of 1.5 mL/min. Headspace samples of 1 µL were injected at 1:10 split. The gas chromatography conditions used were as follows: the oven temperature was at 60 °C, held for 1 min, ramp 40 °C/min until 120 °C, and held for 3.5 min. The C_3_F_8_ was eluted at 1.2 min. The samples were analyzed in selected ion monitoring (SIM) mode using electron impact ionization. A *m/z* of 169 (M-19) was used in the analyses. The ion dwell time was set to 10 msec. The C_3_F_8_ peak was verified by the NIST MS spectra database. A calibration curve was made using different concentrations of C_3_F_8_ prepared by diluting pure C_3_F_8_ gas with air (0–1% *v/v*) and filling a headspace vial containing 1 mL of PBS. The linear regression plot obtained from the standards was used to quantify the C_3_F_8_ gas volume generated by NBs and MBs.

### 2.5. Quantification of Bubble Population Acoustic Response

The in vitro echogenicity of NBs and MBs was determined for six concentrations of bubbles with an equivalent gas volume concentration. The bubble solutions were placed in a custom-made 1.5% (*w/v*) agarose mold with a triple channel (L × W × H per channel = 5 × 3 × 6 mm) [44]. The agarose phantom was fixed over a 12 MHz linear array transducer and imaged using a clinical ultrasound scanner (AplioXG SSA-790A, Toshiba Medical Imaging Systems, Otawara-Shi, Japan). The system acquisition parameters were set to contrast harmonic imaging (CHI) with a 12.0 MHz harmonic frequency, 0.10 mechanical index (MI), 65 dB dynamic range, and 70 dB gain. The ultrasound images were acquired at an imaging frame rate of 1 frame per second (fps) and the intensity per frame was analyzed using the built-in software (CHI-Q). The same region of interest (ROI) was drawn for each sample and the signal decay over an 8 min time period for each sample concentration was quantified using exported data. The signal enhancement by the bubbles was calculated by normalizing the measured signal with respect to that of the agarose phantom. Three trials were performed for each sample analyzed.

### 2.6. Theoretical Calculation of Bubble Gas Volume

The theoretical gas volume based on the RMM, NTA, and Coulter counter measurements was quantified by using the number of different bubble populations of different sizes to obtain a distribution of volumes that were then each multiplied by the number of events for each population. The theoretical amount of perfluorocarbon gas in a bubble was calculated based on the volume of a sphere, assuming that the thickness of the particle shell is 2.5 nm (since we expected that the shell was a lipid monolayer and the typical lipid bilayer thickness is 5 nm). The formula to calculate the theoretical gas volume (in nanoliters, nL) of a single bubble with diameter, *d*, in nanometers (nm), is given in the equation below:

$$V=\frac{4}{3}\pi {\left(\frac{d-5}{2}\right)}^{3}\times \left(1\times 10^{-15}\right)$$

Multiplying the volume per bubble with the concentration will yield the total gas volume of the sample.

## 3. Results and Discussion

Perfluorocarbons (PFC) are ideal for use as clinical bubble ultrasound contrast agents because they are highly stable, do not degrade inside the body (they are non-metabolized), and are highly biocompatible and non-toxic. PFC biological safety is demonstrated by their use in many other biomedical applications, such as PFC emulsions as oxygen carriers (blood substitutes) and as drug delivery systems [18,45,46]. In this study, we quantified the amount of perfluoropropane (C_3_F_8_) gas released by lipid-shelled nanobubbles and microbubbles, using headspace gas chromatography/mass spectrometry (GC/MS), following a previously published report [25,38]. The quantified gas volume was then compared to theoretical measurements using the sizes and concentrations derived from the RMM, NTA, and Coulter counter. The bubbles were isolated using centrifugation at specific speeds from 30–300 rcf following the activation of the lipid solution, and different volumes of the bubbles were transferred to headspace vials. The vials were then sonicated at 50 °C, which ensured the complete destruction of the bubbles, releasing C_3_F_8_ completely to the headspace of the vial because of its insolubility with the aqueous matrix [38]. The amount of C_3_F_8_ in both standards and samples was quantified by the fragment ion *m/z* = 169, which was monitored in SIM mode.

**Figure 1:**
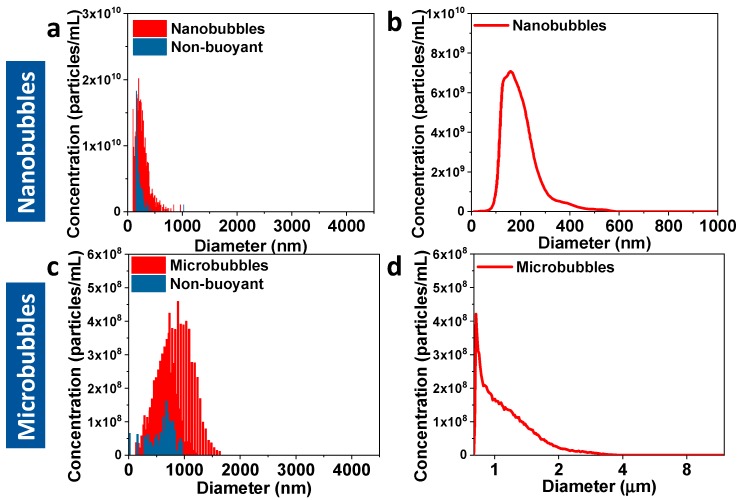
Nanobubbles and microbubbles were characterized using different techniques: (**a**,**c**) resonant mass measurement (RMM), (**b**) nanoparticle tracking analysis (NTA), and (**d**) Coulter counter.

**Figure 2:**
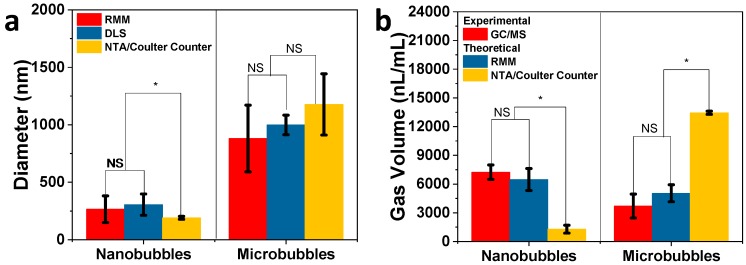
(**a**) Mean bubble size determined using resonant mass measurement (RMM), dynamic light scattering (DLS), nanoparticle tracking analysis (NTA), and Coulter counter. (**b**) Comparison of C_3_F_8_ gas volume determined experimentally using headspace gas chromatography/mass spectrometry (GC/MS) and theoretically predicted based on RMM, NTA, and Coulter counter size and concentration measurements shown in (**a**) and Table 1, respectively. Statistical comparison of size and gas volume of nanobubbles and microbubbles were performed using one-way ANOVA (*p* < 0.05). An asterisk * indicates that the difference of the means is statistically significantly different, while NS means they are not statistically significantly different.

The nanobubbles and microbubbles were comprehensively characterized using RMM, DLS, NTA, and the Coulter counter (Figure 1 and Figure S1), which served as the basis for theoretical gas volume calculations. For nanobubbles (Figure 1a and Figure S1a), it was observed that RMM (265 ± 116 nm) and DLS (305 ± 93 nm) gave comparable size measurements, while NTA (191 ± 13 nm) reported a smaller size for the nanobubbles (Figure 1b). The mean sizes measured with these three techniques show that the result from NTA is significantly different from RMM and DLS (Figure 2a). The smaller size obtained from NTA is most likely because of its lower limit of detection (10 nm) compared to RMM, but, unlike the RMM and DLS, it can only measure up to 1 µm [31]. RMM has the advantage of being able to distinguish between bubbles and non-buoyant particles, from which no other instrument is capable of. On the other hand, NTA has a higher throughput compared to RMM in terms of the sampling measurement. For the microbubble samples, the RMM (881 ± 290 nm), DLS (999 ± 85 nm), and Coulter counter (1175 ± 405 nm) showed comparable size measurements and the means were not significantly different from one another (Figure 1c,d and Figure S1b, Figure 2a). The total gas volume calculated from the RMM, NTA, and Coulter counter measurements were performed by obtaining the gas volume for each bin size and adding them all up to obtain the total gas volume, which reflected more precisely the contribution of each bubble size/population to the total gas volume of the sample. For both the nanobubble and microbubble samples, the RMM showed a comparable concentration measurement with both the NTA and Coulter counter (Table 1). However, it is important to note that the NTA and Coulter counter does not distinguish between bubbles and non-buoyant particles. The RMM measurements reported only the bubble population but the NTA and Coulter counter measurements included both bubbles and non-buoyant particles. While it is rarely pointed out, due to limitations in characterization techniques, non-buoyant particles are unavoidably present in a bubble sample due to the way the bubbles are generated (Figure 1a,c). The difference in size measurements among the three techniques (RMM/DLS vs. Coulter counter) for microbubble characterization was smaller compared to the difference in nanobubble size measurements using RMM/DLS and NTA. This is most likely because the microbubbles have an average size of 1 μm, which is bigger than the limit of detection (400 nm) of the Coulter counter (Figure 2a). A comparison of the experimental C_3_F_8_ gas volume of nanobubbles measured using headspace GC/MS and RMM showed that the two are highly correlated. However, there was a large difference between the predicted and measured gas volume when NTA was used for measurement (Figure 2b). This may be a result of a high number of small but non-buoyant particles counted by the NTA. The NTA, despite having a comparable particle concentration as RMM, is not able distinguish between buoyant and non-buoyant particles. For microbubbles, the predicted gas volume using size measurements based on the RMM and Coulter counter were 0.4- and 2.7-fold higher, respectively, compared to the GC/MS volume measurements. This is most likely due to the microbubbles being less stable compared to the nanobubbles, leading to a rapid gas loss during the sampling process. The Coulter counter showed a much larger deviation than RMM for microbubbles compared to the experimental headspace GC/MS measurement, most likely because of having a relatively high limit of detection, which skewed the population distribution to larger bubbles. Hence, statistical analysis (one-way ANOVA, *p* < 0.05) shows that the predicted and experimentally determined gas volumes between RMM and headspace GC/MS are comparable, but the Coulter counter is statistically significantly different to both (Figure 2b). This suggests that RMM is a more reliable measurement technique for both bubble concentration measurement and, consequently, the calculation of gas volume. A limitation of the RMM during the measurement of nanobubbles is that there may be rare MBs that the technique cannot capture as a function of the limitation of the size range that the nanosensor can detect, which can contribute to the perfluoropropane gas volume of the sample and the ultrasound signal enhancement, as well. Accordingly, a secondary modality, with a broader measurement range, such as the RMM with the microsensor, was used to supplement the RMM nanosensor results, and confirmed the lack of microbubbles present in the sample (Figure S2).

**Figure 3:**
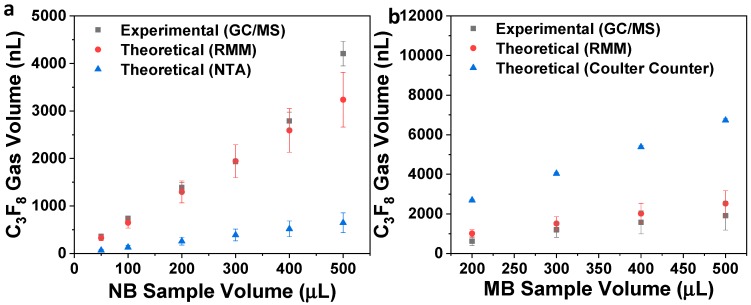
Comparison of perfluoropropane (C_3_F_8_) gas volume determined experimentally using headspace gas chromatography/mass spectrometry (GC/MS) and theoretically predicted based on RMM and Coulter counter size and concentration measurements at different experimental bubble sampling volumes for (**a**) nanobubbles and (**b**) microbubbles.

We also evaluated the effect of the bubble sample volume on the gas volume quantified via the headspace GC/MS. The nanobubble sample volumes from 50–500 μL showed comparable gas volume headspace GC/MS measurements compared to the theoretically predicted measurements using size measurements from RMM, but significantly different from that when using size measurements from the NTA (Figure 3a). For the sample volumes from 200–500 μL, the experimental gas volume quantification of microbubbles showed larger standard deviations (Figure 3b). This could be due to the instability of the microbubble sample, especially during the sampling process, when it was aliquoted to the headspace vial. Microbubbles were less stable in this experimental setup because of their greater buoyancy, which carried them more rapidly to the air-water interface, leading to faster dissipation and inconsistent sampling. In comparison, due to the relatively neutral buoyancy of nanobubbles, their sampling was much more consistent. Due to the very high sensitivity of headspace GC/MS measurements, minute changes in bubble composition were detected (such as loss of gas due to bubble destruction). At these different sampling volumes, the RMM size distributions, while still divergent from experimental GC/MS measurements, appear to be more reliable as a basis for theoretical gas volume calculations compared to the Coulter counter (Figure 3 and Figure S3). In addition, a linear curve fitting of the experimental GC/MS gas volume results of nanobubbles (*y* = 6.3*x* + 79.5) and microbubbles (*y* = 4.6*x* − 288.7) yielded r^2^ values of 0.9867 and 0.9821, respectively. This indicates that there is a linear relationship between bubble sample volume and gas volume experimentally determined from headspace GC/MS.

**Figure 4:**
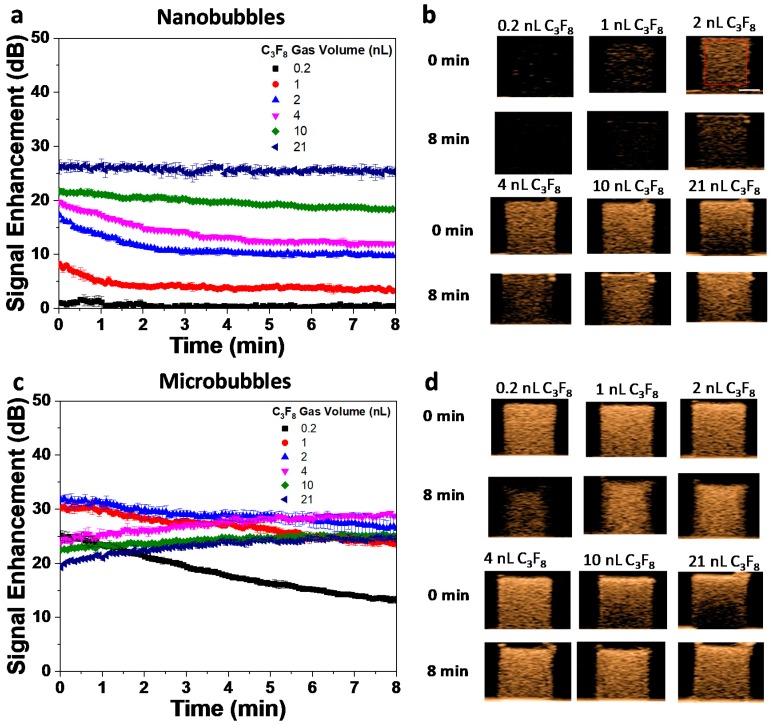
Stability under exposure to ultrasound (f: 12 MHz, MI: 0.1, and frame rate: 1 fps) of the as-prepared bubbles was evaluated at different C_3_F_8_ gas volume concentrations for (**a**,**b**) nanobubbles and (**c**,**d**) microbubbles. The open square in red (b, 2 nL C_3_F_8_) shows a representative region of interest (ROI) used to analyze the time intensity curves. Representative scale bar (b, 2 nL C_3_F_8_) for all the images: 2 mm.

The acoustic activity of the bubbles was evaluated in vitro with varying gas volume concentrations (0.2–21 nL C_3_F_8_) using a clinical ultrasound unit (AplioXG SSA-790A, Toshiba Medical Imaging Systems, Otawara-Shi, Japan) at a frequency of 12 MHz, a mechanical index (MI) of 0.10, and a frame rate of 1 frame per second. The nanobubbles showed increasing signal enhancement and stability over time (progressive reduction in decay rate) with an increasing gas volume and bubble concentration (Figure 4a,b). The same trend was observed for microbubbles but, in addition, there was an increase in signal enhancement over time for the three highest bubble gas volumes evaluated (4, 10, 21 nL) (Figure 4c). Due to the high bubble concentration used, some shadowing at these conditions was observed (Figure 4d). This has been previously reported [47] and can be attributed to acoustic attenuation in the medium and other factors, which include increased bubble–bubble interactions, decreasing the signal strength [25,48]. In both cases, the nanobubbles and microbubbles showed excellent stability under in vitro ultrasound exposure. At equivalent gas volumes, the microbubbles had a higher ultrasound signal enhancement compared to nanobubbles. This is likely due to two factors: (1) an increased scatter from larger-sized bubbles, and (2) potential microbubble resonance. The nanobubbles showed good echogenic signal stability at higher gas volumes, and the minimum gas volume for a detectable signal was significantly higher for nanobubbles compared to microbubbles. For the same gas volume, the scattering strength of the solutions of MBs and NBs with the same shell composition depends on their (a) size, (b) number density, and (c) interaction between bubbles at higher concentrations. It should also be noted that since the images were acquired in harmonic mode, non-linear bubble oscillations significantly contributed to the solution echogenicity. The signal from MBs and NBs for ultrasound enhanced imaging depends on all the above factors. The results illustrate the importance of accurate gas volume calculations, as the activity of both MBs and NBs (but primarily NBs) depends heavily on this parameter.

The acoustic activity of the bubbles was evaluated in vitro with varying gas volume concentrations (0.2–21 nL C_3_F_8_) using a clinical ultrasound unit (AplioXG SSA-790A, Toshiba Medical Imaging Systems, Otawara-Shi, Japan) at a frequency of 12 MHz, a mechanical index (MI) of 0.10, and a frame rate of 1 frame per second. The nanobubbles showed increasing signal enhancement and stability over time (progressive reduction in decay rate) with an increasing gas volume and bubble concentration (Figure 4a,b). The same trend was observed for microbubbles but, in addition, there was an increase in signal enhancement over time for the three highest bubble gas volumes evaluated (4, 10, 21 nL) (Figure 4c). Due to the high bubble concentration used, some shadowing at these conditions was observed (Figure 4d). This has been previously reported [47] and can be attributed to acoustic attenuation in the medium and other factors, which include increased bubble–bubble interactions, decreasing the signal strength [25,48]. In both cases, the nanobubbles and microbubbles showed excellent stability under in vitro ultrasound exposure. At equivalent gas volumes, the microbubbles had a higher ultrasound signal enhancement compared to nanobubbles. This is likely due to two factors: (1) an increased scatter from larger-sized bubbles, and (2) potential microbubble resonance. The nanobubbles showed good echogenic signal stability at higher gas volumes, and the minimum gas volume for a detectable signal was significantly higher for nanobubbles compared to microbubbles. For the same gas volume, the scattering strength of the solutions of MBs and NBs with the same shell composition depends on their (a) size, (b) number density, and (c) interaction between bubbles at higher concentrations. It should also be noted that since the images were acquired in harmonic mode, non-linear bubble oscillations significantly contributed to the solution echogenicity. The signal from MBs and NBs for ultrasound enhanced imaging depends on all the above factors. The results illustrate the importance of accurate gas volume calculations, as the activity of both MBs and NBs (but primarily NBs) depends heavily on this parameter.

## 4. Conclusions

Our work reports, for the first time, the validation of gas volume predictions in echogenic nanobubbles and microbubbles calculated based on size and concentration measurements from four different measurement techniques: RMM, DLS, NTA, and the Coulter counter. Gas volume is an important parameter in ultrasound contrast agent research. The in vitro and in vivo acoustic activity of bubbles are usually compared under normalized gas volume conditions. In this study, we provide evidence that RMM may be more robust and provide accurate measurements of bubble size and concentration compared to measurements based on the NTA and Coulter counter because of its ability to distinguish between bubbles and non-buoyant particles. Because of technique limitations, the Coulter counter tended to overestimate bubble size, resulting in a less accurate theoretical gas volume calculation compared to experimental measurements. Our results show a comparable gas volume obtained between theoretical calculations, using size distributions based on RMM and experimental headspace GC/MS measurements, with RMM having an average of 10% less gas volume for NBs and 27% more gas volume for MBs at the different sampling volumes measured. In contrast, the predicted gas volume using NTA was 82% lower than that of the experimental GC/MS measurements of NBs, while the Coulter counter concentration predicted gas volume that was 2.7-fold higher for MBs compared to the headspace GC/MS results. Experimentally, the nanobubbles showed more reliable measurements at different sampling volumes compared to microbubbles due to better stability, and the fact that the effects were easily detected by the highly sensitive headspace GC/MS instrument.

## Figures and Tables

**Table 1:** Comparison of bubble concentrations measured using RMM, NTA, and Coulter counter.

Sample	RMM (Bubbles/mL)	NTA/Coulter Counter (Particles/mL)
Nanobubbles	4.07 ± 0.11 × 10^11^	4.16 ± 0.28 × 10^11^
Microbubbles	1.08 ± 0.23 × 10^10^	1.14 ± 0.05 × 10^10^

## Supplementary Materials

The following are available online at https://www.mdpi.com/1999-4923/12/3/208/s1, Figure S1: Dynamic light scattering (DLS) size measurement of (a) nanobubbles and (b) microbubbles. Figure S2: (a) Nanobubbles characterized with RMM using a microsensor. Figure S3: Comparison of perfluoropropane (C_3_F_8_) gas volume determined experimentally using headspace GC/MS and theoretically predicted based on RMM, NTA, and Coulter counter. Figure S4: Representative US image of bubble solutions placed in a custom-made 1.5% (*w/v*) agarose mold with a triple channel and a schematic of the phantom and ultrasound transducer location.
