# Supplementary material for: Theoretical and Experimental Gas Volume Quantification of Micro- and Nanobubble Ultrasound Contrast Agents
Source: Pharmaceutics. 2020 Mar 1;12(3):208. doi: 10.3390/pharmaceutics12030208 (PMC7150797; doi:10.3390/pharmaceutics12030208)
Supplement: Supplementary file 1 [file pharmaceutics-12-00208-s001.pdf]

pharmaceutics

MDPI

Supplementary Materials: Theoretical and Experimental Gas Volume Quantification of Micro- and Nanobubble Ultrasound Contrast Agents

Eric C. Abenojar, Ilya Bederman, Al C. de Leon, Jinle Zhu, Judith Hadley, Michael C. Kolios, and Agata A. Exner

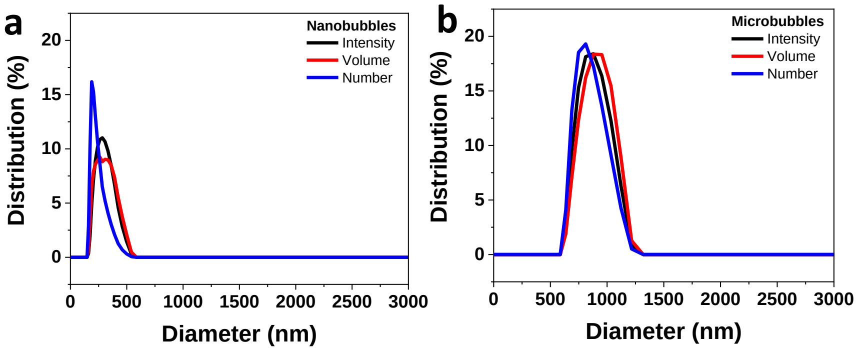

Figure S1. Dynamic light scattering (DLS) size measurement of (a) nanobubbles and (b) microbubbles.

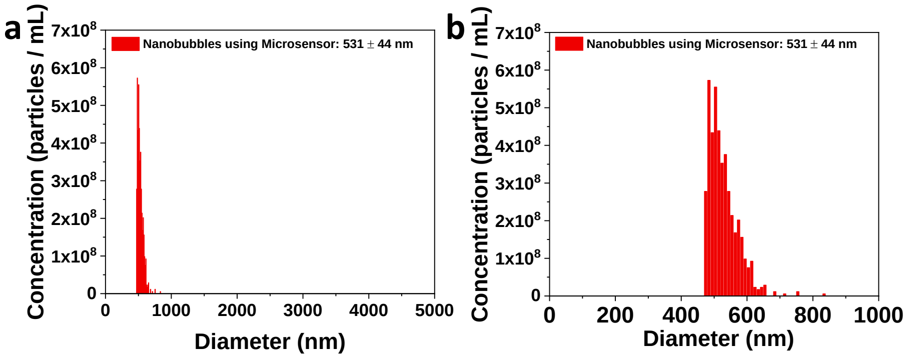

Figure S2. (a) Nanobubbles characterized with RMM using a microsensor showing the absence of any microbubbles (≥ 1 μm) and (b) shows a zoom-in version of the x-axis in (a) indicating the absence of any microbubbles.

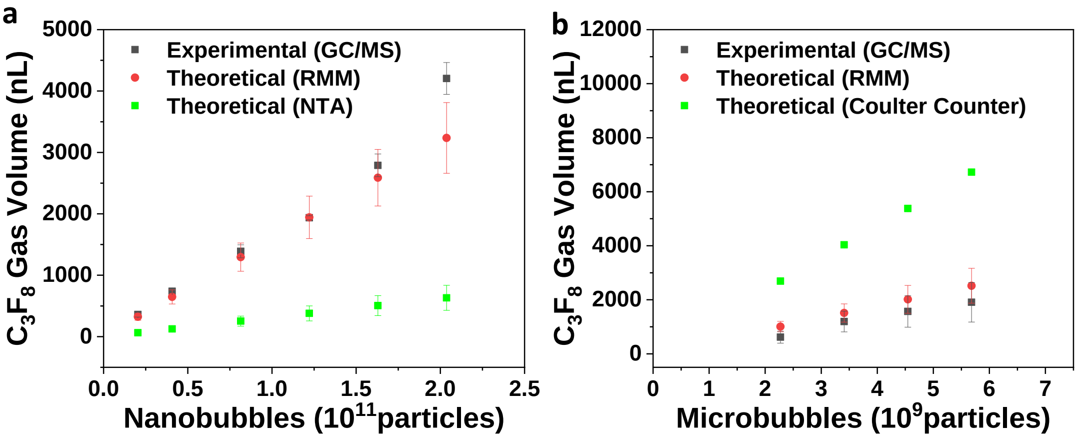

Figure S3. Comparison of perfluoropropane (C_3F_8) gas volume determined experimentally using headspace GC/MS and theoretically predicted based on RMM, NTA, and Coulter counter size and concentration measurements. Bubble concentration was normalized to that of RMM.

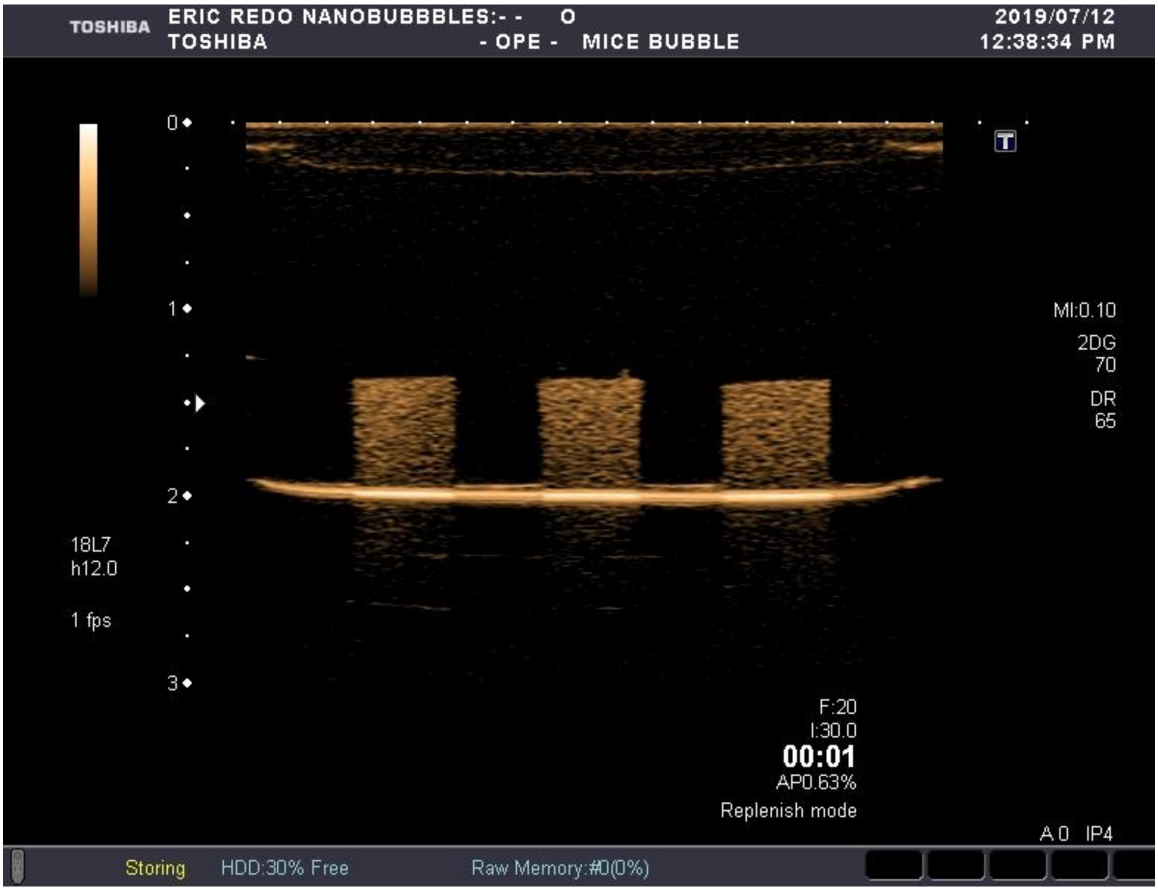

Figure S4. Representative US image of bubble solutions placed in a custom-made 1.5% (w/v) agarose mold with a triple channel (L x W x H per channel = 5 x 3 x 6 mm). A schematic of the phantom and ultrasound transducer location has been shown in a previous report [1].

Reference:

1. Abenojar, E.C.; Nittayacharn, P.; de Leon, A.C.; Perera, R.; Wang, Y.; Bederman, I.; Exner, A.A. Effect of Bubble Concentration on the in Vitro and in Vivo Performance of Highly Stable Lipid Shell-Stabilized Micro- and Nanoscale Ultrasound Contrast Agents. *Langmuir* **2019**, *35*, 10192–10202.
